# Segment Any Leaf 3D: A Zero-Shot 3D Leaf Instance Segmentation Method Based on Multi-View Images

**DOI:** 10.3390/s25020526

**Published:** 2025-01-17

**Authors:** Yunlong Wang, Zhiyong Zhang

**Affiliations:** School of Electronic and Communication Engineering, Sun Yat-sen University, Shenzhen 518000, China; wangylong8@mail2.sysu.edu.cn

**Keywords:** plant phenotyping, instance segmentation, RGB sensors, zero-shot segmentation, multi-view stereo

## Abstract

Exploring the relationships between plant phenotypes and genetic information requires advanced phenotypic analysis techniques for precise characterization. However, the diversity and variability of plant morphology challenge existing methods, which often fail to generalize across species and require extensive annotated data, especially for 3D datasets. This paper proposes a zero-shot 3D leaf instance segmentation method using RGB sensors. It extends the 2D segmentation model SAM (Segment Anything Model) to 3D through a multi-view strategy. RGB image sequences captured from multiple viewpoints are used to reconstruct 3D plant point clouds via multi-view stereo. HQ-SAM (High-Quality Segment Anything Model) segments leaves in 2D, and the segmentation is mapped to the 3D point cloud. An incremental fusion method based on confidence scores aggregates results from different views into a final output. Evaluated on a custom peanut seedling dataset, the method achieved point-level precision, recall, and F1 scores over 0.9 and object-level mIoU and precision above 0.75 under two IoU thresholds. The results show that the method achieves state-of-the-art segmentation quality while offering zero-shot capability and generalizability, demonstrating significant potential in plant phenotyping.

## 1. Introduction

Plant phenotypes are a comprehensive manifestation of all physical, physiological, and biochemical characteristics and traits that reflect the structure, composition, and growth and development processes of plants, influenced jointly by their genotype and the environment [1]. Understanding plant phenotypic characteristics and traits is a significant topic in biological research, as it helps uncover the complex interactions between the genome and environmental factors that shape plant phenotypes [2]. This, in turn, enables the prediction of phenotypes from genotypes, facilitating the effective selection and improvement of crop varieties [3], which is crucial in the pursuit of more efficient and sustainable agriculture. The goal of plant phenotyping is to understand and describe the morphological characteristics and traits of plants under different environmental conditions, which involves the quantitative measurement and evaluation of these traits. Previous quantitative analysis methods primarily relied on manual, destructive measurements, which were limited in both speed and accuracy and were time-consuming and labor-intensive. Consequently, the development of non-destructive and efficient high-throughput phenotyping technologies has become a key research focus across various fields.

Two-dimensional digital phenotyping, while widely used for its high throughput and efficiency [4], has several limitations. It lacks depth information, which results in incomplete representations of plant structures, especially when dealing with occlusions or overlapping parts. This makes the accurate measurement of three-dimensional traits, such as volume and spatial distribution, challenging. Additionally, 2D methods struggle with complex plant morphologies and are highly sensitive to environmental factors like lighting and angle, which can distort results. These limitations hinder the precise analysis of plant traits and their reliable measurement. In contrast, 3D phenotyping technologies capture depth data, allowing for more accurate measurements of plant size, volume, and spatial structure. By addressing the challenges of occlusion, overlap, and irregular morphology, 3D systems provide a more comprehensive and reliable approach to plant phenotyping, making them essential for advancing plant analysis in phenotypic research.

While it is relatively straightforward to obtain overall plant phenotypic information (such as plant height and convex hull volume), finer measurements at the organ or part level require organ-level segmentation to precisely isolate individual organs, such as fruits or leaves. In 3D phenotypic analysis, segmenting the model into distinct plant organs is a crucial yet challenging step for achieving accurate measurements of specific plant parts. Nevertheless, researchers have developed various organ-level segmentation methods, which have yielded promising results.

### 1.1. Traditional Phenotyping Methods

Traditional organ-level phenotyping methods primarily rely on clustering algorithms and region-growing algorithms. The basic principle of clustering methods is to group points in a point cloud into different clusters based on their features, such as spatial position, color, and normal vectors. Xia et al. [5] employed the mean-shift clustering algorithm to segment plant leaves from background elements in depth images. By analyzing the vegetation characteristics of candidate segments generated through mean shift, plant leaves were extracted from the natural background. Moriondo et al. [6] utilized structure from motion (SfM) technology to generate point cloud data for olive tree canopies. They combined spatial scale and color features, which were emphasized in the data, with a random forest classifier to effectively segment the point cloud into distinct plant structures, such as stems and leaves. Zermas et al. [7] implemented an algorithm known as RAIN to segment maize plants. However, this method struggles to perform effectively when dealing with canopies that are densely foliated. Miao et al. [8] introduced an automated method for segmenting stems and leaves in maize by leveraging point cloud data. This approach begins by extracting the skeleton of the maize point cloud, followed by the application of topological and morphological techniques to categorize and quantify various plant organs.

The region-growing algorithm is a segmentation method based on point cloud similarity and neighborhood connectivity. Its fundamental principle involves starting from one or more initial seed points and progressively merging adjacent points that meet specific similarity criteria into the corresponding region until the entire point cloud is segmented into multiple clusters. Liu et al. [9] developed a maize leaf segmentation algorithm that combines the Laplacian operator with the region-growing algorithm. This method first uses the Laplacian operator to generate the plant skeleton [10] and then applies the region-growing algorithm [11] to divide the point cloud into multiple clusters based on the curvature and angular variation of the normal vectors along the organ surface. Miao, Wen et al. [12] applied a median-based region-growing algorithm [13] to achieve semi-automatic segmentation of maize seedlings.

The parameter selection in traditional methods typically depends on the specific structure of the point cloud, which means that the processing parameters suitable for one plant may not be applicable to another. The effectiveness of a given method is influenced by factors such as the plant’s morphology and the quality of its 3D representation [14].

### 1.2. Deep Learning-Based Phenotyping Methods

Deep learning approaches have become highly effective for segmenting 2D images [15,16]. However, most of these segmentation techniques are tailored for structured data and do not perform well with unstructured data, such as 3D point clouds.

PointNet [17] was the first to directly use point cloud data as input for neural networks, accomplishing tasks such as object classification and segmentation. Building upon PointNet, Y. Li et al. [18] developed a point cloud segmentation system specifically for automatically segmenting maize stems and leaves. However, PointNet struggles to capture local structures, limiting its ability to recognize fine-grained patterns and making it difficult to apply in more complex scenarios. In response, Qi et al. [19] proposed PointNet++, whose multi-scale neural network structure recursively applies PointNet to hierarchically partition the input point sets, somewhat addressing the limitations of PointNet. Heiwolt et al. [20] applied the improved PointNet++ architecture to the segmentation of tomato plants, successfully using the network to directly predict the pointwise semantic information of soil, leaves, and stems from the point cloud data. Both Shen et al. [21] and Hao et al. [22] combined the PointNet++ architecture with an improved region-growing algorithm to achieve enhanced local feature extraction. They developed an organ segmentation network specifically designed for cotton seedlings, enabling accurate extraction of their 3D phenotypic structural information.

The irregularity and unordered nature of point cloud data prevent the direct application of convolutional operators designed for regular data. The emergence of PointCNN [23] and PointConv [24] extended classic CNN architectures to point cloud data. Ao et al. [25] used PointCNN to segment the stems and leaves of individual maize plants in field environments, overcoming key challenges in extracting organ-level phenotypic traits relevant to plant organ segmentation. Gong et al. [26] proposed Panicle-3D, a network that achieves higher segmentation accuracy and faster convergence than PointConv, and applied it to the point clouds of rice panicles. However, this method requires large amounts of annotated data for training. Subsequently, Jin et al. [27] proposed a voxel-based CNN (VCNN) as an alternative convolution method to extract features, applying it to semantic and leaf instance segmentation of LiDAR point clouds from 3000 maize plants. Zarei et al. [28] developed a leaf instance segmentation system specifically designed for sorghum crops by integrating the EdgeConv architecture with the DBSCAN algorithm. This system was successfully applied in large-scale field environments. Li et al. [29] introduced PlantNet, a dual-function point cloud segmentation network and the first architecture capable of handling multiple plant species, applying it to tobacco, tomato, and sorghum plants. Building on this, they enhanced the network’s feature extraction and fusion modules, naming the improved network PSegNet [30], which achieved better results than PlantNet.

Another approach is the multi-view representation method, which projects 3D representations onto multiple 2D images and then uses 2D image segmentation networks to obtain segmentation results. The main challenges of this technique lie in determining viewpoint information and mapping the segmentation results back from 2D to 3D space. Shi et al. [31] used ten grayscale cameras from different angles to observe plants, capturing clear segmentation images of the plant and background, and generated a 3D point cloud of the plant using a shape-from-silhouette reconstruction method. They trained a fully convolutional network (FCN) based on an improved VGG-16 and a Mask R-CNN network to achieve semantic and instance segmentation, respectively, and used a voting strategy to merge the segmentation results from the ten 2D images into a 3D point cloud of tomato seedlings. However, this voting scheme is based on the assumption that each point in the point cloud is visible to all ten cameras. Due to occlusions, this assumption is difficult to maintain with complex plants or scenes.

Despite the various methods and improvements, several key challenges remain in plant phenotyping extraction. These challenges include the difficulty of applying the methods in complex scenes with occlusions, the reliance on high-quality annotated 3D plant datasets, and the generalization ability of methods across diverse plant segmentation tasks. To overcome the limitations of existing methods, this paper proposes a zero-shot 3D leaf instance segmentation algorithm based on deep learning and multi-view stereo (MVS). This approach extends the generalization and zero-shot capabilities of the 2D segmentation model SAM into 3D space and introduces a novel fusion method to incrementally merge multiple instance segmentation results. For this paper, the main contributions are as follows:

- It introduces a zero-shot, training-free 3D leaf segmentation method based on multi-view images, which offers accuracy superior to that of traditional methods and overcomes the deep learning requirement for large amounts of training data.
- An incremental merging method based on confidence scores is proposed to integrate 3D point cloud instance segmentation results from multiple viewpoints.
- It provides an efficient, cost-effective, and scalable solution for phenotypic analysis in agriculture.

## 2. Materials and Methods

### 2.1. Overview of the Method

**Figure 1:**
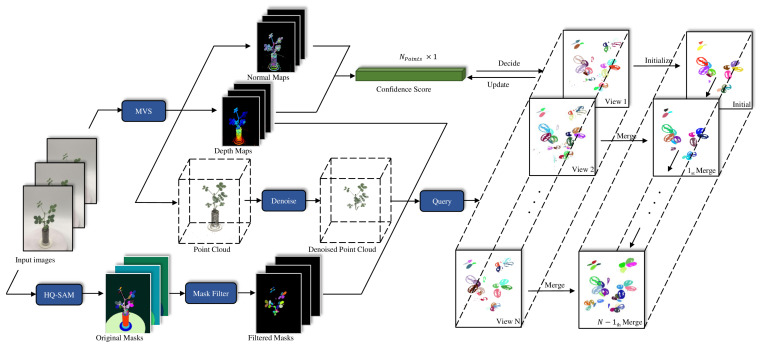
Overview of method. The input image sequence undergoes multi-view stereo (MVS) reconstruction, which is based on feature point matching algorithms to align images from multiple viewpoints and reconstruct a 3D point cloud. HQ-SAM-based image segmentation is then applied to extract instance segmentation from each image. The 2D grouping information is mapped into 3D space through a querying process, and the results are incrementally merged to generate the final instance segmentation.

Figure 1 illustrates the proposed method, which processes the input image sequence data using two key modules: MVS 3D reconstruction and 2D leaf instance segmentation. In the 3D reconstruction module, camera poses for multiple viewpoints are estimated using structure from motion (SfM), followed by depth and normal estimation and the fusion of these data to generate a 3D point cloud. Meanwhile, the multi-view image sequence is fed into an instance segmentation network based on the Segment Anything Model (SAM) to obtain segmentation masks, which are further refined to produce high-quality leaf masks. Based on the camera pose information estimated from SfM, the segmentation mask for each point is queried across the images to obtain a coarse 3D instance segmentation. Finally, confidence scores are generated by combining depth and normal information, which are used for the incremental fusion of 3D point cloud groups.

The following sections provide a detailed overview of the apparatus used for image sequence collection, the MVS-based 3D reconstruction process (Section 2.2), and the 2D leaf instance segmentation approach (Section 2.3). Additionally, we explain the point cloud query method for transferring 2D grouping information into 3D (Section 2.4) and outline the incremental merging method for instance segmentation results (Section 2.5). Finally, the evaluation methods are presented (Section 2.6).

### 2.2. Data Collection and 3D Reconstruction

The image sequence collected in this study includes 16 peanut seedling samples with a growth period ranging from 14 to 28 days. The multi-view image sequence capture setup we used is similar to that used by Wang et al. [32], where a remote-controlled, motorized smart electric turntable is used to rotate the plant. An annular light source is fixed at the top of the studio to reduce shadows. The plant is placed on the turntable in the studio, and an Apple iPhone 8 camera (Apple Inc., Cupertino, CA, USA) is focused on the center of the plant. The camera is adjusted to an appropriate height with a top-down angle of approximately 30–45 degrees. A sequence of 32 images, each with a resolution of 3024 × 4032 pixels, is captured at uniform intervals of 11.25 degrees within a 360-degree range to cover the entire surface of the plant, ensuring high-quality 3D reconstruction. The entire acquisition process for 32 images is completed within approximately 2 min.

Subsequently, the open-source MVS reconstruction software COLMAP (v3.10) [33] is used for plant point cloud reconstruction. In COLMAP, feature point matching is performed on multi-view images using SfM to estimate the camera pose information of these images. This process plays a crucial role in querying the mapping from 3D point clouds to 2D image pixels. Then, dense reconstruction is applied to estimate multi-view depth maps and normal maps, which are fused to generate a dense point cloud. In a standard 3D space, the point cloud model generated using MVS technology is normalized, and the data volume is large, often including irrelevant background noise such as pots and soil. Therefore, preprocessing is required to create a clean 3D model of the plant.

**Figure 2:**
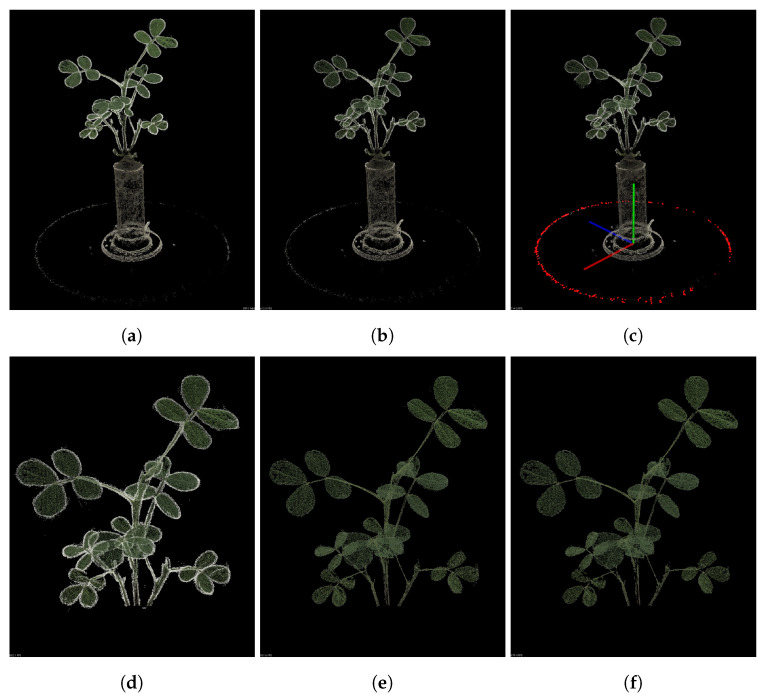
Point cloud denoising process: (**a**) the original point cloud reconstructed using COLMAP; (**b**) the point cloud after voxel downsampling; (**c**) fitting the turntable using RANSAC and adjusting the coordinate system; (**d**) the point cloud after pass-through filtering; (**e**) the point cloud after color filtering; (**f**) the point cloud after statistical filtering.

The point cloud processing workflow is shown in Figure 2. The point cloud coordinate system reconstructed by COLMAP is typically based on the initial camera pose, so a rough correction of the coordinate system is required to align it with the real 3D space. To denormalize the point cloud, a scale factor is computed using a reference object’s actual dimensions. In this study, we extract the point cloud of the rotating platform and apply random sample consensus (RANSAC) for 3D circle fitting to calculate the scale factor. The coordinate system is then fine-tuned based on the RANSAC fitting results. With the adjusted coordinate system, we can easily apply pass-through filtering to remove the plant pot and retain only the stem and leaves of the plant. At this stage, some erroneous points at the edges of the point cloud may still remain, having a similar color to the background. These points are filtered out using an RGB filter. Finally, for scattered outliers, statistical filtering is applied for removal. To ensure the correct application of the estimated camera pose information, the coordinate system is adjusted back to its original state.

### 2.3. Instance Segmentation on Single 2D Image

The segmentation model builds on SAM [34], a highly efficient and versatile image segmentation framework. SAM first processes input images through a ViT-based encoder to extract image features and generate spatial embeddings. A prompt encoder then encodes input information and feeds it to a two-layer transformer-based mask decoder, which generates the final segmentation mask. HQ-SAM [35] enhances SAM’s segmentation quality on fine structures by introducing the HQ-Output Token and Global–Local Feature Fusion. To maintain SAM’s zero-shot capability, the lightweight HQ-Output Token reuses SAM’s mask decoder, with added MLP layers performing pointwise multiplication using fused HQ features.

**Figure 3:**
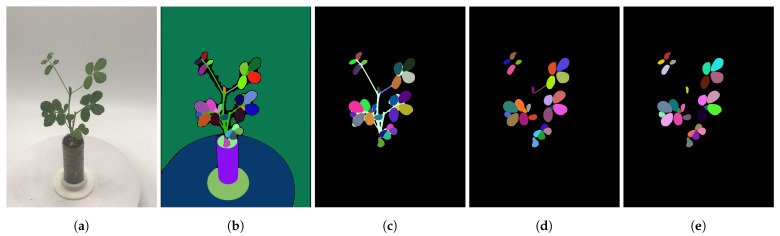
Mask filtering process. (**a**): The original RGB image; (**b**): the automatic masks generated by HQ-SAM; (**c**): the masks after saturation filtering; (**d**): the masks after overlap removal; (**e**): the masks after shape filtering. All samples used the same parameters. The (**b**) process averaged 7.95 s on a GeForce RTX 3090 GPU (NVIDIA, Santa Clara, CA, USA), while the (**c**–**e**) processes averaged 1.15 s, 6.19 s, and 2.50 s on an Intel(R) Xeon(R) Silver 4210 CPU (Intel Corporation, Santa Clara, CA, USA).

In this pipeline, the pretrained ViT-H model automatically generates segmentation masks from 32 multi-view images. Notably, to enhance the inference speed of HQ-SAM, we downsampled the original 3024 × 4032 resolution images to about 1500 × 2000 for use as model input. Since no prompts are used, the generated masks often include many objects that are not of interest. We are only concerned with leaf objects, so further filtering of these masks is required. The processing pipeline, based on Williams et al. [36], is designed to obtain the desired mask objects. It consists of three filtering steps in total (Figure 3):

1. Saturation Filter: The automatically generated masks by HQ-SAM often contain objects other than the plant, such as the background, flowerpot, etc. To isolate the relevant parts of the plant, we calculate the average saturation value for all the pixels within each mask object. By applying threshold-based filtering, we can retain the regions of interest. In our experiment, the image acquisition environment is controlled, with the background consisting of low-saturation white or gray areas. Therefore, a filtering threshold of S > 40 is set to exclude irrelevant masks while retaining leaf masks.
2. Overlap Filter: After the first step, we obtain masks related to the plant itself. However, due to segmentation ambiguities, individual pixels may appear in multiple masks simultaneously. Since we aim for organ-level segmentation rather than whole-plant segmentation, the overlap filter detects overlaps between any two masks by calculating their IoU. We retain the mask with finer granularity.
3. Shape Filter: The remaining masks at this point still include both leaf and stem segments. To remove stem masks, we utilize shape characteristics. Leaves tend to have fuller shapes, while stems are more elongated. We first calculate the area of the minimum enclosing circle for each mask and then compute the mask area. By using the ratio of these two values, we can easily identify and remove masks corresponding to stems.

### 2.4. Segmentation on 3D Point Clouds

In a three-dimensional point cloud $P=\{p_{1},p_{2},\ldots ,p_{N}\}$, the coordinates of a point p are represented as a three-dimensional vector $\left(X_{w},Y_{w},Z_{w}\right)$ in the world coordinate system. To map the grouping information of this point into three-dimensional space, we need to query its instance segmentation mask labels from different viewpoints. This process involves converting coordinates from the world coordinate system to the pixel coordinate system (Equation (1)).

(1) $$\left[\begin{array}{c} u\\ v\\ 1 \end{array}\right]=\frac{1}{Z_{c}}K\left[\begin{array}{cc} R & T\\ 0 & 1 \end{array}\right]\left[\begin{array}{c} X_{w}\\ Y_{w}\\ Z_{w}\\ 1 \end{array}\right]$$

where (u,v) represents the pixel coordinates of point p in a particular image, $\;Z_{c}$ is the depth of point p in the camera coordinate system,K is the 3×3 camera’s intrinsic parameter matrix, and E=[R|T] is the 3×4 camera extrinsic parameter matrix, which includes a 3×3 rotation matrix R and a 3×1 translation matrix T. Both K and E were obtained during the previous COLMAP dense reconstruction process.

For a point on a 2D image, its corresponding range in 3D space is a conical region, known as the view frustum. This characteristic may cause ambiguity in the mapping process, where a single pixel point could correspond to multiple three-dimensional points. Therefore, we need to use the depth map as a reference, ensuring through Equation (2) that points on the pixel do not map to three-dimensional points with mismatched depths.

(2) $$e^{\left(u,v\right)}=\frac{d^{\left(u,v\right)}-Z_{c}^{\left(u,v\right)}}{d^{\left(u,v\right)}}$$

where *d* is the depth on pixel point (u,v). By controlling the threshold of error, ambiguous points can be excluded.

### 2.5. Incremental Merging

#### 2.5.1. Preprocessing

Given the 3D masks derived in the previous subsection, we introduce an incremental merging approach to merge the mask predictions from different frames. Specifically, we denote the point cloud from two adjacent images by $X=\left\{\left(k_{x}^{1},l_{x}^{1}\right),\left(k_{x}^{2},l_{x}^{2}\right),\ldots ,\left(k_{x}^{m},l_{x}^{m}\right)\right\}$ and $Y=\left\{\left(k_{y}^{1},l_{y}^{1}\right),\left(k_{y}^{2},l_{y}^{2}\right),\ldots ,\left(k_{y}^{n},l_{y}^{n}\right)\right\}$, where *m* and *n* are the numbers of points in *X* and *Y*, *k* is the point index in the original point cloud, and *l* is the grouping label generated in the previous subsection. In our representation, each point in the original point cloud $P=\left\{\left(l_{x}^{1},l_{y}^{1}\right),\left(l_{x}^{2},l_{y}^{2}\right),\ldots ,\left(l_{x}^{N},l_{y}^{N}\right)\right\}$ is associated with two sets of grouping information from viewpoint *X* and viewpoint *Y*, and N is the number of points in the original point cloud. Based on this, we compute the correspondence mapping *M* between *X* and *Y*. M can be represented as a dictionary that records the point cloud indices under each correspondence, as determined by *P*.

For each mask label $l_{x}$ in *X*, we can use *M* to find the number of points from $l_{x}$ that correspond to labels in *Y*, along with their point cloud indices. As illustrated in $l_{x}:\left\{l_{y}^{1}:\gamma_{1},l_{y}^{2}:\gamma_{2},\ldots ,l_{y}^{n}:\gamma_{n}\right\}$, where the normalized point count γ represents the overlap ratio between $l_{x}$ and the respective labels. Similarly, for each label in *Y*, we can also determine its corresponding situation in *X*. The two overlap ratio tables are denoted by $Q_{x}$ and $Q_{y}$ and will be used in subsequent processing.

#### 2.5.2. Computation of Confidence Score

During the fusion of instance segmentation information from different viewpoints, conflicting results between the two sets of segmentation often arise. In such cases, we need a criterion to determine which viewpoint’s segmentation result should be adopted for the final fusion. Intuitively, we tend to choose the segmentation result with higher quality. The quality of image segmentation is often influenced by various factors, such as the shooting angle and image clarity. Therefore, we propose a method for calculating confidence scores based on the camera viewpoint and imaging distance, which is used to assess the reliability or quality of the segmentation masks.

Based on the depth maps and normal maps obtained in the previous Section 2.2, we can retrieve the normal vector $\left(n_{x},n_{y},n_{z}\right)$ and depth *d* at each point $\left(x_{p},y_{p},z_{p}\right)$. Additionally, using the camera’s extrinsic matrix, the camera position $\left(x_{c},y_{c},z_{c}\right)$ can be determined, allowing us to compute the viewpoint vector $\vec{v}=\left(x_{p}-x_{c},y_{p}-y_{c},z_{p}-z_{c}\right)$. Thus, the angle between the viewpoint vector and the normal vector at that point is $\cos\left(\phi \right)=\frac{\vec{v}\cdot \vec{n}}{|\vec{v}\left|\right|\vec{n}|}$.

**Figure 4:**
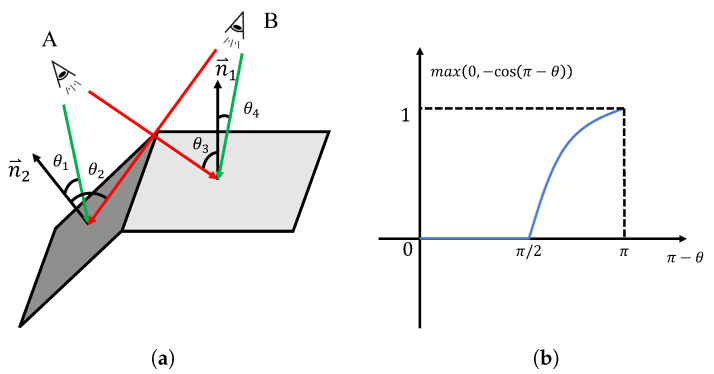
Illustration of different views. (**a**): The angular relationship between different viewpoints and the pixel plane. A and B are two different camera viewpoints. Green indicates that the viewpoint is better for the surface at this location, while red represents a worse viewpoint; (**b**): the calculation function for the viewpoint factor.

As shown in Figure 4a, the closer the complementary angle θ between the viewpoint vector and the normal vector is to 0 radians, the more perpendicular the line of sight is to the object plane, resulting in better viewpoint quality. Conversely, when θ approaches $\frac{\pi }{2}$ radians, the line of sight becomes parallel to the object plane, leading to lower segmentation quality. If $\theta \in (\frac{\pi }{2},\pi )$, it indicates that the viewpoint is on the backside of the target plane, and the segmentation result is disregarded. Based on this idea, the confidence score is calculated by Equation (3).

(3) $$s=\lambda \max\left(0,-\cos\phi \right)+\left(1-\lambda \right)d_{n}$$

Here, λ∈(0,1) is a scaling factor used to control the weighting of viewpoint and distance in the decision process, and $d_{n}$ is the normalized depth. We calculate the confidence score for each point in every viewpoint, resulting in an $N_{points}\times 1$ array.

#### 2.5.3. Process Merging

Based on the mapping relationships, all cases can be divided into three categories: $\left(l_{x},l_{y}\right)$, $\left(l_{x},-1\right)$, and $\left(-1,l_{y}\right)$, where −1 indicates that the point lacks grouping information in either *X* or *Y*. Typically, we first handle unmatched cases like $\left(l_{x},-1\right)$ and $\left(-1,l_{y}\right)$. For unmatched points, various scenarios may arise. When the majority of points in a group are unmatched, we treat this group as a separate new group and directly add all points in this group as a new group to the target point cloud segmentation. These points inherit the original confidence scores.

The remaining unmerged points are in the form of (x,y), and this scenario can be further divided into two cases. The first scenario occurs when two masks from different viewpoints represent the same group of information, referred to as a matching group. To identify these matching groups, we need to calculate the IoU between each pair of masks $l_{x}^{i}$ and $l_{y}^{j}$, meaning that both groups have a significantly high overlap ratio $\gamma_{x}^{\left(i,j\right)}$, $\gamma_{y}^{\left(i,j\right)}$ in $Q_{x}$ and $Q_{y}$. All points from $l_{x}^{i}$ in *X* and $l_{y}^{j}$ in *Y* are merged into one group. Since the group information is mutually validated across both viewpoints, their confidence scores are summed point by point, indicating that these points have higher reliability.

**Figure 5:**
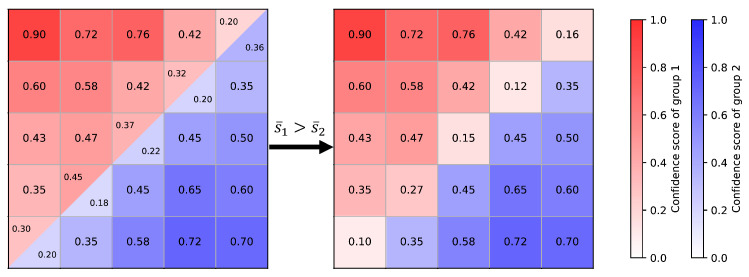
Illustration of merging conflict groups.

In the second case, there is a conflict between the grouping results from the two viewpoints, meaning that $l_{x}^{i}$ and $l_{y}^{j}$ only partially overlap, or one mask’s point set is a proper subset of the other. In this situation, the average confidence scores of the two masks need to be compared. As shown in Figure 5, there is a partial overlap between the point sets from $l_{x}$ and $l_{y}$, requiring the labels to be determined for the overlapping region. We first calculate the average confidence scores of the two groups by Equation (4).

(4) $$\bar{s}=\frac{1}{N}\sum_{i=1}^{N}s_{i}$$

If the average confidence score of $l_{x}$ is higher than that of $l_{y}$, the labels for the overlapping region are assigned to $l_{x}$; otherwise, they are assigned to $l_{y}$. The update rule for the confidence scores is as follows:

(5) $$s_{new}=\left\{\begin{array}{cc} \left|s_{x}-s_{y}\right|, & \mathrm{if}\;\mathrm{overlapping}\\ s_{pre}, & \mathrm{if}\;\mathrm{non}\text{-}\mathrm{overlapping} \end{array}\right.$$

non-overlapping points retain their original confidence scores, while for overlapping points, the confidence scores are updated by taking the absolute difference.

### 2.6. Performance Evaluation

To validate the effectiveness of the approach in this paper, performance evaluations were conducted at both the point and object levels. We used CloudCompare [37] to manually segment the plant samples, assigning a unique label to each instance to generate the ground truth. For the point-level evaluation, true positives, false positives, and false negatives were determined by comparing the predicted mask $S^{pred}$ on a point-by-point basis. True positives refer to the points that appear in both the predicted and ground-truth masks, with their count determined by Equation (6). False positives refer to points that the method incorrectly predicts but which do not appear in the ground-truth mask, as shown in Equation (7). On the other hand, false negatives are points that are included in the ground-truth mask but are overlooked by the prediction, with their count defined by Equation (8).

(6) $$\mathrm{TP}=∥S^{pred}\cap S^{gt}∥$$

(7) $$\mathrm{FP}=∥S^{pred}∥-\mathrm{TP}$$

(8) $$\mathrm{FN}=∥S^{gt}∥-\mathrm{TP}$$

The point-level segmentation results were then assessed using precision, recall, and F1 score. Precision indicates the proportion of correctly detected pixels or objects (Equation (9)), while recall measures the proportion of the plant parts correctly segmented (Equation (10)). The F1 score reflects the balance between precision and recall (Equation (11)). The closer these metrics are to 1, the better the performance.

(9) $$\mathrm{Precision}=\frac{\mathrm{TP}}{\mathrm{TP}+\mathrm{FP}}$$

(10) $$\mathrm{Recall}=\frac{\mathrm{TP}}{\mathrm{TP}+\mathrm{FN}}$$

(11) $$F1=2\cdot \frac{\mathrm{Precision}\times \mathrm{Recall}}{\mathrm{Precision}+\mathrm{Recall}}$$

In the object-level evaluation, the focus is on assessing the overall segmentation results rather than individual points. For each group in the segmentation, the overlap between the ground-truth and predicted masks, quantified by the Intersection over Union (IoU), is determined using Equation (12), and the average value is obtained. The mean IoU (mIoU) reflects the overall accuracy of plant-part segmentation, with values ranging from 0 to 1, where a value closer to 1 indicates better segmentation results:

(12) $$\mathrm{mIoU}=\frac{1}{N}\sum_{i=1}^{N}\frac{∥S_{i}^{pred}\cap S_{i}^{gt}∥}{∥S_{i}^{pred}\cup S_{i}^{gt}∥}$$

AP (Equation (13)) is computed from the precision–recall curve, which measures the model’s average precision across different IoU thresholds:

(13) $$\mathrm{AP}=\sum_{i=2}^{N}P\left(i\right)\times \left(R\left(i\right)-R\left(i-1\right)\right)$$

In subsequent experiments, the AP will be calculated at IoU thresholds of 50 and 75. These thresholds indicate that a predicted group is only considered correct if its IoU with the ground truth exceeds the specified threshold.

To further assess the number of leaves after segmentation, we compare the number of predicted groups with the ground-truth number of leaves. The estimation error is quantified using the Coefficient of Determination $R^{2}$, the Mean Absolute Percentage Error (MAPE), and the Root Mean Square Error (RMSE). $R^{2}$ (Equation (14)) is used to measure the goodness of fit of the model, representing the linear correlation between the model’s output and the actual data, with a value range from 0 to 1. The closer the value is to 1, the better the model fits the data. MAPE (Equation (15)) is a dimensionless error metric that measures the relative error between the predicted and actual values, helping to quantify the model’s performance consistency across different scales. In leaf count estimation, RMSE (Equation (16)) is used to evaluate the absolute error of the model, and it is particularly sensitive to large prediction errors, clearly reflecting the impact of significant deviations in the estimation process. A lower RMSE indicates that the predicted results are closer to the actual values. Since RMSE squares the errors, it is more sensitive to outliers, amplifying the impact of larger errors.

(14) $$R^{2}=1-\frac{\sum {\left(y_{i}-{\hat{y}}_{i}\right)}^{2}}{\sum {\left(y_{i}-\bar{y}\right)}^{2}}$$

(15) $$\mathrm{MAPE}=\frac{1}{N}\sum_{i=1}^{N}\left|\frac{y_{i}-{\hat{y}}_{i}}{y_{i}}\right|\times 100\%$$

(16) $$\mathrm{RMSE}=\sqrt{\frac{1}{N}\sum_{i=1}^{N}{\left(y_{i}-{\hat{y}}_{i}\right)}^{2}}$$

where $y_{i}$ represents the ground truth, and ${\hat{y}}_{i}$ represents the estimated value.

## 3. Results

### 3.1. Instance Segmentation Evaluation

**Figure 6:**
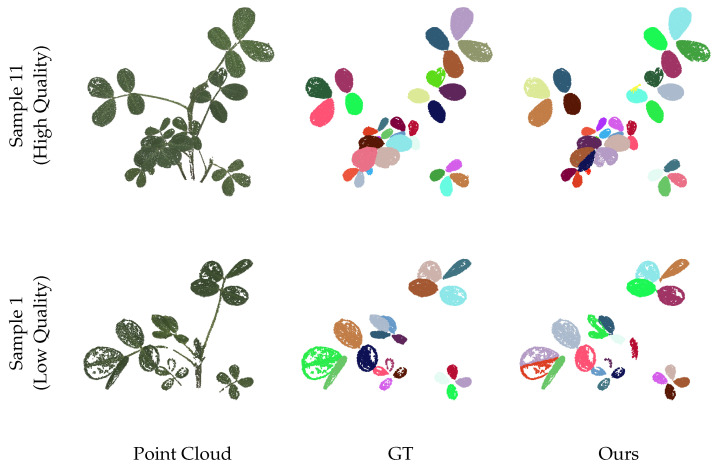
Visualization of leaf instance segmentation. The point cloud quality of sample 1 is low, and there are many holes present. Sample 11 has higher point cloud quality.

Figure 6 presents several examples of leaf instance segmentation on 3D point clouds using the methods described in Section 2. From the dataset, we selected two plants with varying morphologies and point cloud qualities and visualized the segmentation results alongside the ground truth. Different colors represent each distinct leaf instance. It is worth noting that the weak texture characteristics of the leaf surfaces result in holes in the 3D reconstruction, which pose challenges for leaf segmentation and test the robustness of our method. The quantitative evaluation of the two samples is shown in Table 1. Even in the case of samples with poor point cloud quality, our method still achieves promising results, demonstrating strong robustness.

The projection depth error threshold *e* and the confidence score scaling factor λ play crucial roles in influencing the final outcomes of leaf segmentation. As mentioned in Section 2.4, the choice of threshold is crucial for eliminating ambiguous points when mapping 2D pixels to 3D space. A smaller threshold may result in spatial points that should correspond to pixel points not being properly assigned grouping information, leading to a decrease in recall and classification accuracy. Conversely, a larger threshold may incorporate incorrect points into the group, reducing precision. Therefore, selecting an appropriate threshold is critical. The scaling factor primarily determines whether viewpoint factors or distance factors should take precedence when resolving conflicting groups. Our processing is completed in a normalized point cloud, which ensures consistency and automation in parameter selection. There is no need to fine-tune each sample.

In Table 2, we observe how point-level and object-level performance metrics change with different error thresholds. Consistent with our analysis, when e<0.1%, precision is the highest, but recall is the lowest. When e<2.0%, recall is higher, but precision is lower. At a threshold of e<0.5%, the F1 score reaches its maximum, achieving a balance between precision and recall. At this threshold, the object-level segmentation results are also optimal.

In Table 3, we set the error threshold to e<0.5% to evaluate the performance of different scale factors λ. The results indicate that at a scale factor of λ=0.3, the point-level segmentation results are the best, while at a scale factor of λ=0.9, the object-level segmentation results are optimal. However, unlike the evaluation of the error threshold, the variations in scale factors have a negligible impact on the segmentation results. This is because when the angle between the leaf plane and the viewpoint is suboptimal, the mask of the leaf in the 2D image appears elongated, resembling the shape of a stem, making it prone to being filtered out. Meanwhile, the masks that are not filtered correspond to a limited range of angles between the leaf plane and the viewpoint, causing the differences in confidence scores among the different masks to be very subtle.

Overall, for the task of leaf segmentation, our method demonstrates highly accurate performance across all point-level metrics (precision, recall, and F1 score). Our precision consistently exceeds 0.95, indicating very high accuracy. However, the recall is relatively low compared to precision. This is because, during the process of merging instance information, pre-filtering is applied to remove outlier points from the instance masks. Although these points still remain in the point cloud, their instance labels are removed, causing some points to be excluded from the merging process. This increases the number of false negatives and, consequently, lowers the recall. The F1 score reflects a good balance between precision and recall. Comparing point-level accuracy with object-level AP values, it can be seen that while the precision is high, the accuracy in object recognition is relatively low, indicating that object-level prediction may face greater challenges.

### 3.2. Comparison with Other Methods

In this section, we compare the method proposed in this paper with existing mainstream leaf instance segmentation methods. Among these, DBSCAN, mean shift, and region growing are traditional segmentation methods that do not require dataset training. In contrast, PlantNet and PSegNet are deep learning networks specifically designed for plant leaf segmentation tasks. To the best of our knowledge, PSegNet remains the current state of the art (SOTA) for this task. We applied extensive data augmentation to our collected peanut dataset and used the preprocessing code provided by PSegNet to standardize the input point clouds to a size of 4096 points, increasing its size 100-fold, resulting in a total of 1600 point clouds with 4096 points each. The dataset was then split into training and testing sets with a 5:3 ratio, which is close to the 2:1 split used in PSegNet, as it is not possible to divide 16 samples by a 2:1 ratio. Using the recommended parameter configurations, we trained the networks for 100 epochs and computed the evaluation metrics as the mean values over the test set.

**Figure 7:**
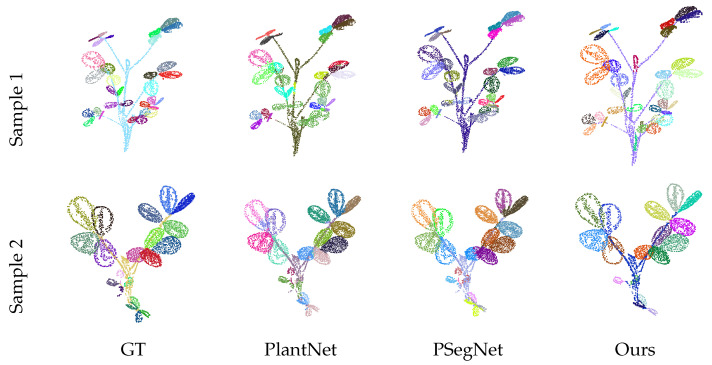
The qualitative instance segmentation comparison between three methods. For consistency with the visualizations of PlantNet and PSegNet, the results from our method were downsampled to a similar point cloud size of approximately 4096 points, and stem areas were included.

Table 4 presents a quantitative comparison of the six methods for the instance segmentation task. Our method significantly outperforms traditional approaches and exhibits complementary strengths compared to the SOTA model across various metrics. PSegNet excels in precision but demonstrates relatively low recall and F1 scores. In the object-level evaluation, PSegNet slightly outperforms our method in less stringent metrics ($AP_{50}$). However, our method achieves the best performance under the strictest threshold ($AP_{75}$) and demonstrates the highest mIoU. Notably, our method requires no training. The qualitative comparison for the instance segmentation task is shown in Figure 7. The samples in the figure demonstrate that our method outperforms the specialized plant leaf segmentation models, PlantNet and PSegNet, both in overall performance and in capturing finer details.

### 3.3. Estimation of Leaf Number

**Figure 8:**
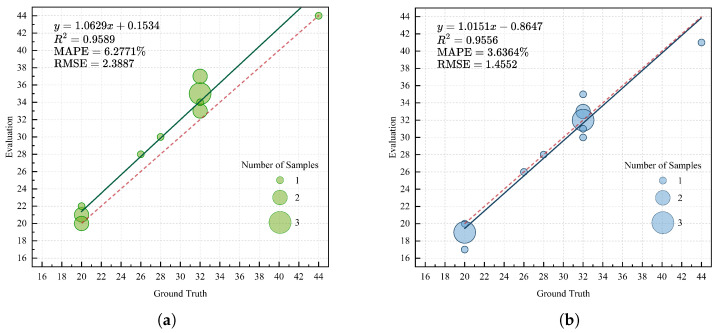
Leaf instance segmentation quantity analysis: (**a**) Analysis of the instance count with buds compared to the ground truth; (**b**) analysis of the instance count without buds compared to the ground truth. The size of each bubble in the bubble chart represents the sample size, and the red dashed line indicates the reference fitting line y=x.

In Figure 8, we analyze the results of the instance segmentation group counts, which correspond to the estimation of leaf numbers. It is noteworthy that our samples include some newly emerging leaves or buds that are about to unfold, which are detected by our method. Strictly speaking, this does not constitute an incorrect segmentation result; however, our aim is to disregard such leaves. Therefore, we discuss the two scenarios of including and excluding buds in Figure 8a,b, respectively. When buds are included, the MAPE and RMSE are 6.28% and 2.39, respectively. When buds are excluded, the MAPE and RMSE are 3.63% and 1.46, respectively. In both cases, the MAPE is less than 10%, falling within the acceptable range for morphological-scale phenotyping [38]. After excluding the buds, the linear weighted fit almost overlaps with the reference fit, indicating that the proposed method can perform high-quality segmentation of leaf objects.

### 3.4. Generalization Ability

Leveraging the strong generalization capability of the SAM model, our proposed method also exhibits excellent generalizability, meeting the requirements of segmentation tasks in diverse scenarios. However, due to the lack of plant sample datasets, we conducted generalization tests on the DTU dataset [39]. The DTU dataset is specifically designed for MVS evaluation, comprising 124 scenes, each captured from 49 or 64 viewpoints, resulting in a corresponding number of RGB images with a resolution of 1600 × 1200. The DTU scenes feature a wide variety of objects and are captured under diverse lighting conditions. We selected several representative scenes from the DTU dataset to perform instance segmentation experiments.

**Figure 9:**
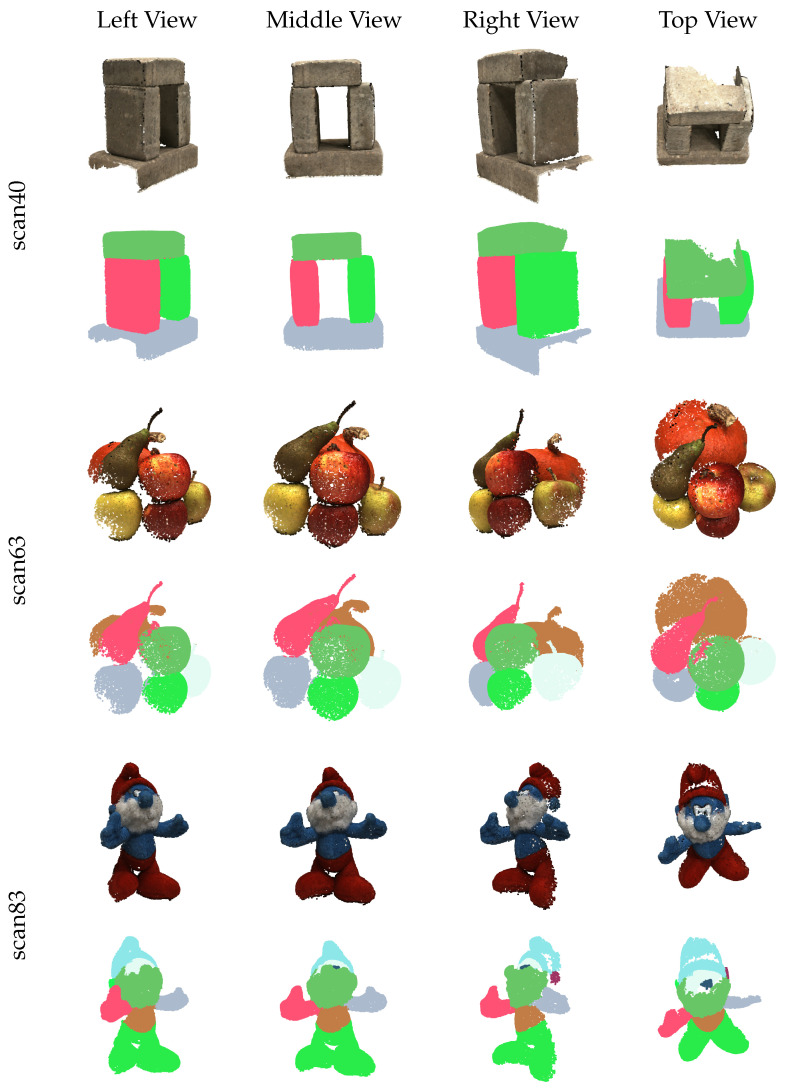
Visualization of instance segmentation on DTU dataset.

The qualitative segmentation results on the DTU dataset are shown in Figure 9. These scenes differ significantly from plant-related scenarios, yet our method achieves remarkable performance in these cases. This demonstrates the robust generalization capability of our approach, which can effectively meet the segmentation requirements across diverse scenarios.

## 4. Discussion

### 4.1. Effectiveness of the Proposed Method

The effectiveness of our method primarily stems from two modules: the SAM module and the incremental fusion module. The SAM module serves as the cornerstone of the entire pipeline, providing the foundation for the zero-shot capability and generalization of our method. SAM has been trained on the large-scale SA-1B dataset, which includes over 1 billion automatically generated masks and 11 million images [34]. As a result, it demonstrates exceptional zero-shot generalization performance on new datasets. The quality of instance segmentation is heavily influenced by the accuracy of 2D segmentation in each viewpoint. In subsequent sections, we analyze errors produced by our method, most of which originate from inaccuracies in 2D segmentation by the SAM module.

**Figure 10:**
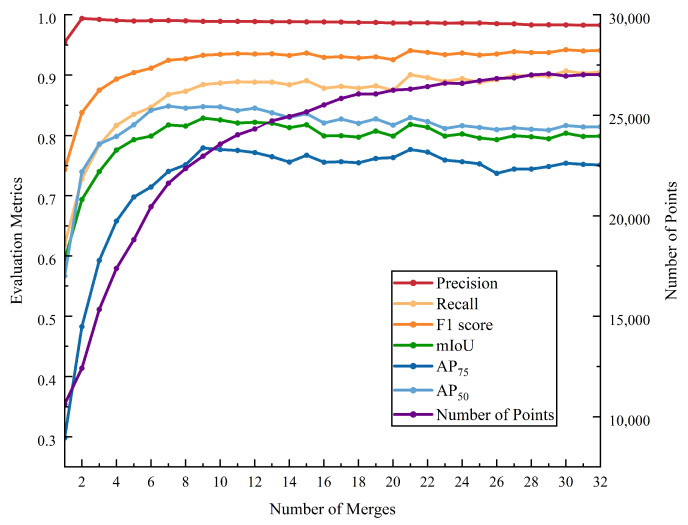
The variations in metrics and point cloud quantity with the number of merges.

The incremental fusion module is a critical step for aggregating multiple point cloud instance segmentation results. Single 2D segmentations are inevitably affected by factors such as occlusion and viewpoint, leading to segmentation errors that propagate when projected into 3D point clouds. Incremental fusion minimizes these errors by continuously refining the segmentation results. As shown in Figure 10, we conducted experiments on how quantitative metrics change with the number of fusion iterations. With an increasing number of fusions, nearly all evaluation metrics show an upward trend, especially precision, recall, and F1 score, indicating the positive impact of fusion on model performance. In the early stages of fusion, all metrics improve steadily; however, as the process approaches its limit, performance gains become marginal, suggesting that the model is nearing its optimal performance. The range of changes in ${\mathrm{AP}}_{75}$ is smaller compared to ${\mathrm{AP}}_{50}$, indicating that improving model performance under high IoU thresholds is relatively more challenging. Slight fluctuations in the later stages of fusion are mainly due to minor performance losses caused by newly added unoptimized point clouds.

In summary, the generalization and zero-shot capabilities of our method are attributed to the SAM module’s pretraining on large-scale datasets. Meanwhile, the incremental fusion module effectively corrects errors in 2D segmentation caused by insufficient information, thereby significantly enhancing overall performance.

### 4.2. Error Analysis

To better understand the performance and the limitations of the systems, errors in 3D instance segmentation are examined in four specific categories. Table 5 provides a comprehensive explanation of the definitions and underlying reasons for each of the four error categories. The error rate for each category is determined by calculating the ratio of instances exhibiting that specific error to the total number of leaf instances.

Over-segmentation and under-segmentation both reduce the IoU and AP. Over-segmentation introduces additional false positives, decreasing the overlap between predicted and actual regions, thus lowering segmentation quality and AP, especially at higher IoU thresholds. Under-segmentation, on the other hand, causes the model to miss certain objects, which reduces recall and further decreases both IoU and AP. While missed detections do not introduce false positives, they positively affect precision. Overlapping segmentation primarily impacts recall, leading to its decline.

The causes of these errors are varied and can be attributed to several factors. First, errors from the 2D SAM segmentation are a major contributor, as the accuracy of our method largely depends on the segmentation results from SAM in 2D images, which form the foundation of our approach. In the process of mask filtering, we adopted a strategy of preserving smaller masks, which can also lead to the over-segmentation of masks. Second, errors introduced during the MVS process also impact the segmentation results. For instance, when the quality of the reconstructed leaves is poor, some small leaf instances may be mistakenly filtered out as noise. Additionally, the depth estimation across different viewpoints is not always consistent. When there are significant depth estimation errors from multiple viewpoints, artifacts may appear on the leaves, leading to segmentation inaccuracies. Finally, errors in camera pose estimation can also affect the mapping of 2D segmentation results into the 3D space. As mentioned in previous sections, the setting of the depth error threshold can also influence the final results to some extent.

### 4.3. Future Works

The proposed incremental merging method enables fast and accurate fusion of multi-view 3D point cloud instance segmentation results. However, the method is not without flaws. Qualitative analysis has explained some of the errors and the mechanisms behind them, such as over-segmentation, under-segmentation, missed detections, and overlapping segmentation. In future work, we aim to improve the method in the following ways: (a) using a more accurate 2D image segmentation network or fine-tuning SAM’s pretrained parameters to better adapt to plant leaf segmentation tasks; (b) trying to use other shape descriptors, such as aspect ratio and roundness, because the minimum circumcircle and mask area ratio may not be applicable to all shapes, especially for leaves that are not completely circular or for stems with irregular shapes; (c) employing 3D reconstruction methods with better weak texture feature extraction and matching capabilities to improve the quality of plant point cloud models; (d) leveraging deep learning-based depth estimation methods, such as the MVS-Net series [40], which have demonstrated superior depth estimation accuracy compared to traditional methods, potentially enhancing algorithm performance; and (e) replacing the MVS reconstruction process with RGB-D cameras, which can significantly improve speed and quality, though at the cost of expensive equipment.

## 5. Conclusions

This paper presents a deep learning-based multi-view 3D instance segmentation method for plant leaves, extending the zero-shot and generalization capabilities of the SAM model to 3D. By incrementally merging segmentation results from multiple 2D viewpoints onto a 3D point cloud, the method achieves high accuracy without requiring data training. On a peanut seedling dataset, the method demonstrated strong performance, achieving point-level precision, recall, and F1 scores of 0.983, 0.910, and 0.944, respectively, and an object-level mIoU of 0.798, with accuracy scores of ${\mathrm{AP}}_{75}$ and ${\mathrm{AP}}_{50}$ being 0.751 and 0.811. Its robust generalization makes it applicable to various plant types, growth stages, and even non-plant scenarios using low-cost RGB sensors with minimal hardware requirements.

This approach facilitates non-destructive, detailed organ segmentation, enabling continuous monitoring of plant development and improving phenotypic data acquisition and analysis. By providing more precise phenotypic data, the method holds potential for advancing crop breeding, improving adaptation to environmental changes, and promoting agricultural sustainability.

## Figures and Tables

**Table 1:** Quantitative evaluation of 3D instance segmentation results under different point cloud qualities.

Samples	Point-Level			Object-Level		
	Precision	Recall	F1 score	mIoU	${\mathrm{AP}}_{75}$	${\mathrm{AP}}_{50}$
Sample 1	0.949	**0.943**	**0.947**	0.776	0.748	**0.802**
Sample 11	**0.991**	0.823	0.899	**0.791**	**0.769**	0.791

These data represent the results for a scale factor λ=0.9 and an error threshold e<0.5%. The optimal parameter values are derived from the experiments shown in Table 2 and Table 3, where bold font represents the best metric.

**Table 2:** Quantitative evaluation of 3D instance segmentation results under different depth error thresholds.

Values	Point-Level			Object-Level		
	Precision	Recall	F1 Score	mIoU	${\mathrm{AP}}_{75}$	${\mathrm{AP}}_{50}$
e<0.1%	0.991	0.834	0.903	0.749	0.670	0.780
e<0.3%	0.985	0.895	0.937	0.785	0.742	0.795
e<0.5% *	0.983	0.910	0.944	0.798	0.751	0.811
e<0.7%	0.980	0.904	0.939	0.790	0.749	0.797
e<1.0%	0.975	0.904	0.936	0.791	0.743	0.796
e<1.5%	0.968	0.914	0.939	0.793	0.744	0.792
e<2.0%	0.966	0.899	0.930	0.776	0.721	0.781

These data represent the results for a scale factor λ=0.5, calculated by averaging all samples. Bold font represents the best metric, and * denotes the best value.

**Table 3:** Quantitative evaluation of 3D instance segmentation results under different scale factors λ.

Values	Point-Level			Object-Level		
	Precision	Recall	F1 Score	mIoU	${\mathrm{AP}}_{75}$	${\mathrm{AP}}_{50}$
λ=0.1	0.983	0.907	0.942	0.798	0.751	0.813
λ=0.3	0.983	0.908	0.943	0.801	0.754	0.816
λ=0.5	0.983	0.905	0.941	0.799	0.751	0.814
λ=0.7	0.983	0.906	0.942	0.800	0.753	0.814
λ=0.9 *	0.983	0.907	0.942	0.802	0.756	0.816

These data represent the results for an error threshold e<0.5%, calculated by averaging all samples. Bold font represents the best metric, and * denotes the best value.

**Table 4:** Quantitative comparison of instance segmentation performance of six methods.

Methods	Training-Free	Point-Level			Object-Level		
		Precision	Recall	F1 Score	mIoU	${\mathrm{AP}}_{75}$	${\mathrm{AP}}_{50}$
DBSCAN	✓	0.712	0.536	0.544	0.320	0.122	0.264
Mean shift	✓	0.567	0.891	0.684	0.402	0.163	0.339
Region growing	✓	0.787	0.630	0.693	0.414	0.280	0.437
PlantNet	✗	0.996	0.707	0.822	0.655	0.357	0.713
PSegNet	✗	**0.997**	0.821	0.897	0.750	0.580	**0.813**
Ours	✓	0.983	**0.910**	**0.944**	**0.798**	**0.751**	0.811

Bold font represents the best metric.

**Table 5:** Four different error categories.

Categories	Definitions	Reasons	Ratio
Over-segmentation	Splitting a single target object into multiple unnecessary parts.	2D SAM segmentation error; Error in leaf mask filtering.	1.98%
Under-segmentation	Multiple independent objects were mistakenly merged into one entity.	2D SAM segmentation error; Error in leaf mask filtering.	1.10%
Missed detection	An instance that should have been segmented but was not correctly detected and classified as an instance.	2D SAM segmentation error; Error in leaf mask filtering; Low point cloud quality; Depth estimation error.	3.74%
Overlapping segmentation	Points belonging to a single instance are assigned to multiple instances.	Low point cloud quality; Depth estimation error; Camera pose estimation error.	1.32%

## Data Availability

The data and the code are available upon request.

## Abbreviations

The following abbreviations are used in this manuscript:

2D	Two-dimensional
3D	Three-dimensional
AP	Average precision
CNN	Convolutional neural network
FCN	Fully convolutional network
FN	False negative
FP	False positive
HQ-SAM	High-Quality Segment Anything Model
IoU	Intersection over Union
LiDAR	Light Detection and Ranging
MAPE	Mean Absolute Percentage Error
MLP	Multilayer perceptron
MVS	Multi-view stereo
RAIN	Randomly Intercepted Nodes
RANSAC	Random sample consensus
R-CNN	Recurrent convolutional neural network
RMSE	Root Mean Square Error
SAM	Segment Anything Model

SfM	Structure from motion
TP	True positive
VCNN	Voxel-based convolutional neural network
ViT	Vision Transformer

## References

[1] Pan Y.H. (2015). Analysis of concepts and categories of plant phenome and phenomics. Acta Agron. Sin..

[2] Chen M., Hofestädt R. (2014). Approaches in Integrative Bioinformatics: Towards the Virtual Cell.

[3] Poland J.A., Rife T.W. (2012). Genotyping-by-Sequencing for Plant Breeding and Genetics. Plant Genome.

[4] Li Z., Guo R., Li M., Chen Y., Li G. (2020). A review of computer vision technologies for plant phenotyping. Comput. Electron. Agric..

[5] Xia C., Wang L., Chung B.K., Lee J.M. (2015). In Situ 3D segmentation of individual plant leaves using a RGB-D camera for agricultural automation. Sensors.

[6] Moriondo M., Leolini L., Staglianò N., Argenti G., Trombi G., Brilli L., Dibari C., Leolini C., Bindi M. (2016). Use of digital images to disclose canopy architecture in olive tree. Sci. Hortic..

[7] Zermas D., Morellas V., Mulla D., Papanikolopoulos N. (2020). 3D model processing for high throughput phenotype extraction—The case of corn. Comput. Electron. Agric..

[8] Miao T., Zhu C., Xu T., Yang T., Li N., Zhou Y., Deng H. (2021). Automatic stem-leaf segmentation of maize shoots using three-dimensional point cloud. Comput. Electron. Agric..

[9] Liu F., Song Q., Zhao J., Mao L., Bu H., Hu Y., Zhu X.G. (2021). Canopy occupation volume as an indicator of canopy photosynthetic capacity. New Phytol..

[10] Cao J., Tagliasacchiy A., Olsony M., Zhangy H., Su Z. Point cloud skeletons via Laplacian-based contraction. Proceedings of the SMI 2010—International Conference on Shape Modeling and Applications.

[11] Vosselman G., Rabbani T., Heuvel F.A.V.D., Vosselman G. (2006). Segmentation of Point Clouds Using Smoothness Constraint. Int. Arch. Photogramm. Remote Sens. Spat. Inf. Sci..

[12] Miao T., Wen W., Li Y., Wu S., Zhu C., Guo X. (2021). Label3DMaize: Toolkit for 3D point cloud data annotation of maize shoots. GigaScience.

[13] Jin S., Su Y., Wu F., Pang S., Gao S., Hu T., Liu J., Guo Q. (2019). Stem-Leaf Segmentation and Phenotypic Trait Extraction of Individual Maize Using Terrestrial LiDAR Data. IEEE Trans. Geosci. Remote Sens..

[14] Harandi N., Vandenberghe B., Vankerschaver J., Depuydt S., Messem A.V. (2023). How to make sense of 3D representations for plant phenotyping: A compendium of processing and analysis techniques. Plant Methods.

[15] Bhagat S., Kokare M., Haswani V., Hambarde P., Kamble R. (2022). Eff-UNet++: A novel architecture for plant leaf segmentation and counting. Ecol. Inform..

[16] Carneiro G.A., Magalhães R., Neto A., Sousa J.J., Cunha A. (2021). Grapevine Segmentation in RGB Images using Deep Learning. Procedia Comput. Sci..

[17] Qi C.R., Su H., Mo K., Guibas L.J. (2017). PointNet: Deep Learning on Point Sets for 3D Classification and Segmentation. arXiv.

[18] Li Y., Wen W., Miao T., Wu S., Yu Z., Wang X., Guo X., Zhao C. (2022). Automatic organ-level point cloud segmentation of maize shoots by integrating high-throughput data acquisition and deep learning. Comput. Electron. Agric..

[19] Qi C.R., Yi L., Su H., Guibas L.J. (2017). PointNet++: Deep Hierarchical Feature Learning on Point Sets in a Metric Space. arXiv.

[20] Heiwolt K., Duckett T., Cielniak G. (2021). Deep Semantic Segmentation of 3D Plant Point Clouds. Towards Autonomous Robotic Systems.

[21] Shen J., Wu T., Zhao J., Wu Z., Huang Y., Gao P., Zhang L. (2024). Organ Segmentation and Phenotypic Trait Extraction of Cotton Seedling Point Clouds Based on a 3D Lightweight Network. Agronomy.

[22] Hao H., Wu S., Li Y., Wen W., Fan J., Zhang Y., Zhuang L., Xu L., Li H., Guo X. (2024). Automatic acquisition, analysis and wilting measurement of cotton 3D phenotype based on point cloud. Biosyst. Eng..

[23] Li Y., Bu R., Sun M., Wu W., Di X., Chen B. (2018). PointCNN: Convolution On *X*-Transformed Points. arXiv.

[24] Wu W., Qi Z., Fuxin L. (2020). PointConv: Deep Convolutional Networks on 3D Point Clouds. arXiv.

[25] Ao Z., Wu F., Hu S., Sun Y., Su Y., Guo Q., Xin Q. (2022). Automatic segmentation of stem and leaf components and individual maize plants in field terrestrial LiDAR data using convolutional neural networks. Crop J..

[26] Gong L., Du X., Zhu K., Lin K., Lou Q., Yuan Z., Huang G., Liu C. (2021). Panicle-3D: Efficient Phenotyping Tool for Precise Semantic Segmentation of Rice Panicle Point Cloud. Plant Phenomics.

[27] Jin S., Su Y., Gao S., Wu F., Ma Q., Xu K., Hu T., Liu J., Pang S., Guan H. (2020). Separating the Structural Components of Maize for Field Phenotyping Using Terrestrial LiDAR Data and Deep Convolutional Neural Networks. IEEE Trans. Geosci. Remote Sens..

[28] Zarei A., Li B., Schnable J.C., Lyons E., Pauli D., Barnard K., Benes B. (2024). PlantSegNet: 3D point cloud instance segmentation of nearby plant organs with identical semantics. Comput. Electron. Agric..

[29] Li D., Shi G., Li J., Chen Y., Zhang S., Xiang S., Jin S. (2022). PlantNet: A dual-function point cloud segmentation network for multiple plant species. ISPRS J. Photogramm. Remote Sens..

[30] Li D., Li J., Xiang S., Pan A. (2022). PSegNet: Simultaneous Semantic and Instance Segmentation for Point Clouds of Plants. Plant Phenomics.

[31] Shi W., van de Zedde R., Jiang H., Kootstra G. (2019). Plant-part segmentation using deep learning and multi-view vision. Biosyst. Eng..

[32] Wang R., Liu D., Wang X., Yang H. (2022). Segmentation and measurement of key phenotype for Chinese cabbage sprout using multi-view geometry. Nongye Gongcheng Xuebao/Transactions Chin. Soc. Agric. Eng..

[33] Schonberger J.L., Frahm J.M. Structure-from-Motion Revisited. Proceedings of the 2016 IEEE Conference on Computer Vision and Pattern Recognition (CVPR).

[34] Kirillov A., Mintun E., Ravi N., Mao H., Rolland C., Gustafson L., Xiao T., Whitehead S., Berg A.C., Lo W.Y. (2023). Segment Anything. arXiv.

[35] Ke L., Ye M., Danelljan M., Liu Y., Tai Y.W., Tang C.K., Yu F. (2023). Segment Anything in High Quality. arXiv.

[36] Williams D., Macfarlane F., Britten A. (2024). Leaf only SAM: A segment anything pipeline for zero-shot automated leaf segmentation. Smart Agric. Technol..

[37] Girardeau-Montaut D. (2016). CloudCompare—Open Source Project. https://www.cloudcompare.org/.

[38] Paproki A., Sirault X., Berry S., Furbank R., Fripp J. (2012). A novel mesh processing based technique for 3D plant analysis. BMC Plant Biol..

[39] Jensen R., Dahl A., Vogiatzis G., Tola E., Aanæs H. Large scale multi-view stereopsis evaluation. Proceedings of the 2014 IEEE Conference on Computer Vision and Pattern Recognition.

[40] Cao C., Ren X., Fu Y. (2024). MVSFormer++: Revealing the Devil in Transformer’s Details for Multi-View Stereo. arXiv.

